# Regulatory/modulatory effect of prune essence concentrate on intestinal function and blood lipids

**DOI:** 10.1080/13880209.2017.1285323

**Published:** 2017-02-05

**Authors:** Hui-Fang Chiu, Yun-Chien Huang, Yan-Ying Lu, Yi-Chun Han, You-Cheng Shen, Oksana Golovinskaia, Kamesh Venkatakrishnan, Chin-Kun Wang

**Affiliations:** a Department of Chinese Medicine, Taichung Hospital, Ministry of Health and Well-being, Taichung, Taiwan, ROC;; b School of Nutrition, Chung Shan Medical University, Taichung City, Taiwan, ROC;; c Department of Neurology, Chung Shan Medical University, Taichung City, Taiwan, ROC;; d School of Health Diet and Industry Management, Chung Shan Medical University, Taichung City, Taiwan, ROC;; e ITMO University, Saint-Peterburg, Russia

**Keywords:** Hypercholesterolemic, intestinal microflora, lipid profile, antioxidant capacity

## Abstract

**Context:***Prunus domestica* Linn (Rosaceae) has been considered a functional food, owing to its various pharmacological activities, including antioxidant, anti-inflammatory, antidiabetic and anticancer.

**Objective:** This placebo-controlled, randomized study was framed to check the beneficial activity of prune essence concentrates (PEC) in corroboration with intestinal function and lipid profile in mildly hypercholesterolemic subjects.

**Materials and methods:** Sixty healthy mild hypercholesterolemic subjects were randomly chosen and segregated into three groups as placebo (consume 50 mL of simulated prune drink), PEC I (consume 50 mL of PEC/day) and PEC II (consume 100 mL of PEC/day) for 4 weeks with 2 weeks of follow-up without PEC consumption.

**Results:** Intake of PEC (I and II) for 4 weeks substantially ameliorated (*p* < 0.05) the colony number of *Bifidobacterium* spp. (1.18- and 1.19-fold) and *Lactobacillus* spp. (1.07- and 1.16-fold), but markedly lowered (*p* < 0.05) the colony number of *Clostridium perfringens* (5.97 and 8.35%) and *Escherichia coli* (6.25 and 9.38%). Meanwhile, the total cholesterol (TC; 5.90 and 6.99%) levels and LDL-c (6.68 and 6.53%) were significantly reduced (*p* < 0.05), but no change in other lipid parameters. Whereas, the antioxidant capacity was also concomitantly elevated (*p* < 0.05) upon administration with PEC.

**Discussion and conclusion:** Overall, the results suggest that the use of PEC may positively regulate the intestinal microflora and thereby effectively lower the TC levels and thus act as a hypocholesterolemic agent.

## Introduction

Epidemiological studies have indicated that cardiovascular diseases (CVDs) claim more lives than any other diseases. In Taiwan, CVDs are the second largest contributor to mortality, which accounts for almost 11.4% of all mortality (Teo et al. 2013; Chiu et al. 2015). Hypercholesterolemia is a condition in which serum total cholesterol (TC) and low-density lipoprotein cholesterol (LDL-c) are substantially elevated and thus believed as a dominant risk factor in the pathophysiology of atherosclerosis and subsequent CVDs (Lu et al. 2015). Several researchers highlighted that maintenance of lipid levels in normal level (normocholesterolemia) might reduce the incidence of CVDs. Currently several lipid-lowering drugs are available in the markets, however, they cause several adverse effects, which leads to the quest for functional food/nutraceuticals with hypocholesterolemic efficacy without triggering any adverse effect is increased enormously.

Experimental data suggested that increased intake of fruits and vegetables may cut down the onset of CVDs, due to the presence of photo components (polyphenols; flavonoids), which attributes for its antioxidant and anti-inflammatory properties (Slavin & Lloyd 2012; Chiu et al. 2015). Especially, fruits (juice or dried form) like apple, berries, raisins, apricot, dates, and prunes are reported to lower the incidence of CVDs (Oyebode et al. 2014). Prune is a dried form of plum fruits of *Prunus domestica* Linn (Rosaceae) Prune is usually processed as juice/essence, jam, puree or other prune products. Several studies demonstrated that prune is rich in both soluble and insoluble fibers, simple sugars especially xylo-oligosaccharides, phenolic acid derivatives (chlorogenic acid, neochlorogenic acid, cryptochlorogenic acid, and oligomeric proanthocyanidin) as well as micronutrients like vitamins and minerals (Stacewicz-Sapuntzakis et al. 2001; Putnam et al. 2007). Prunes are reported to exhibit numerous therapeutic properties including antioxidant, antidiabetic, anticancer, as well as cardioprotective, gastroprotective (laxative) and neuroprotective agents. In addition, it is also recommended for treating bone mineral loss and menstrual related disorders (Stacewicz-Sapuntzakis 2013; Igwe & Charlton 2016).

Existing evidence suggests that fruits rich in fibers and oligosaccharides might act as prebiotics (feeds probiotics) and thus promote the growth of the healthy microorganism (intestinal microflora) and thereby enhance various physiological functions (El-Gawad et al. 2005; Scheid et al. 2013). An impressive number of studies indicated that intestinal microflora plays a pivotal role in maintaining human health status by ameliorating nutrient absorption, effectively removing toxins, maintaining normal mucosal immunity, suppressing pathogen colonization and regulating fat metabolism (Alonso & Guarner 2013; Qiao et al. 2014). Prune has been demonstrated to possess several biological activities (especially hypocholesterolemic). However, the link between prune intake with intestinal microbiota and lipid status as well as its association were not conducted until now. Hence, the current trial was carried out to assess whether the consumption of prune essence concentrates (PEC) modulate the intestinal microflora, antioxidant status and lipid profile in mildly hypercholesterolemic subjects.

## Materials and methods

### Commercial PEC and placebo

Both PEC and placebo were provided by Cerebos Pacific Ltd., Singapore, as BRAND InnerShine Prune Essence. The major ingredients of PEC (each bottle) were summarized as follows: 6 g of dietary fiber (pectin), 0.8 g of oligosaccharides (inulin, xylo-oligosaccharides), 1 g of fructose and trace of sorbitol, malic acid, sodium citrate with water (42.1 kcal/bottle). The placebo beverage contained brown sugar, pectin, malic acid, sodium citrate and simulated with prune-washed water. Both the sample bottles looked alike with similar colour, appearance, flavour, size and shape.

### Subjects and study design

Sixty healthy mild hypercholesterolemic subjects (aged between 18 and 53 years; 27 males and 33 females) were recruited by flyers and advertisement posted in public places and Chung Shan Medical University Hospital, Taiwan. The volunteers included in the present randomized, placebo-controlled study based on medical history questionnaires along with dietary habits and vitals were checked to confirm the health status. The main inclusion criteria included mild hypercholesterolemia (serum cholesterol 170–200 mg/dL) without any metabolic disorder, and the exclusion criteria included the history of drinking, smoking, pregnancy, breastfeeding, chronic illness, gastrointestinal disorders, uncontrolled diabetes mellitus, cardiac or renal dysfunction as well as intake of dietary or nutritive supplements and or any other medications. Volunteers were requested to sign a consent form prior to the intervention.

The current study was approved by the Institutional Review Board (IRB) of the Chung Shan Medical University Hospital, Taichung, Taiwan and conducted (CSMUH: CS10056) in accordance with the guidelines laid down in the Declaration of Helsinki and Good Clinical Practice. All the sixty healthy mild hypercholesterolemic subjects were randomly divided (by digital computerized codes) into three groups as placebo (*n* = 20), who were instructed to consume one bottle (50 mL) of simulated drink with prune-washed water, PEC I (test A; *n* = 20), who were requested to consume one bottle (50 mL) of PEC/day (after a meal) and PEC II (test B; *n* = 20), who were requested to consume 2 bottles (100 mL) of PEC/day for 4 weeks. The total experimental period was carried out for seven weeks, out of which first week (run-in period) was for adaptation or stabilization, followed by four weeks as an intervention period (consumption of samples) and lastly two weeks as follow-up period without any consumption of samples. During the intervention, subjects were requested to abide with normal lifestyle and dietary pattern. Anthropometric measurements were done along with collections of both faecal and blood samples. Based on subject records, the average consumption rate of PEC was about 90% at the end of the experiment. During the intervention period, one female subject in the placebo group, as well as one male from PEC (test A) group was excluded because of personal reasons, and hence only 58 subjects completed the study.

### Blood collection

The fasting blood samples (initial, 4th and 6th weeks) were collected in two tubes, one with EDTA coated for plasma and another without anticoagulant for serum preparation. Plasma was separated and used for evaluating various antioxidant indexes. The serum samples were used for determining the lipid profile. All the blood samples were stored at −80 °C until analysis.

### Faecal sample collection and bacterial enumeration

Faecal samples were collected and weighed, then transferred into the sterile plastic anaerobic bag at initial, 2nd, 4th and 6th week to check the major intestinal microflora. One gram of faecal sample was mixed with 99 mL of peptone saline (peptone and sodium chloride) and homogenized (10%) in different dilution ratios with peptone saline in an anaerobic condition. To quantify aerobic and anaerobic microflora, 20 μL of the homogenized faecal sample was plated on acidified different agar medium and incubated for 48 h in aerobic and anaerobic conditions at 37 °C based on different types of bacterial enumeration. Enumeration of *Escherichia coli, Clostridium perfringens, Lactobacillus* spp.*, Bifidobacteria* spp. and total anaerobic bacteria were done on MacConkey agar (Merck, Germany) by the method of Manafi and Kneifel (1989), TSC agar by Harmon et al. (1971), Rogosa SL agar (Merck, Germany) by Rogosa et al. (1951), BIM 25 agar and Brucella agar (Creative Microbiologicals, Taiwan) by Beerens (1991), respectively. After the incubation period, colonies were identified and characterized by Gram staining, cellular morphology, and catalase reaction. The colonies of various bacterial spp. were calculated by using a formula with dilution factor and dry weight of faeces. The colony counts were reported as log_10_ of colony forming units (CFU/g dry weight of faeces).

### Lipid profiles in serum and oxidative indexes in plasma

The total cholesterol (TC), triglyceride (TG) and high-density lipoprotein cholesterol (HDL-c) were measured by commercial lipid profile kits from Roche Diagnostics (Mannheim, Germany). Low-density lipoprotein cholesterol (LDL-c) was calculated using the Friedewald equation. Total antioxidant capacity in plasma was determined by the method of Miller et al. (1993) with a slight alteration. The total TBARS in plasma was measured by reacting with 2-thiobarbituric acid using the Draper and Hadley (1990) method.

### Statistical analysis

The outcomes were expressed as a mean ± standard deviation (SD). The paired *t*-test was used to compare the changes in the same group, and Student’s *t*-test was used to compare the difference between the PEC and placebo groups and the variables were analyzed via one-way ANOVA with a *post hoc* LSD test using Statistical Package for the Social Sciences (SPSS) version 23.0 (IBM, Chicago, IL). *p-*values <0.05 are deemed as statistically significant.

## Results

**Figure 1.**
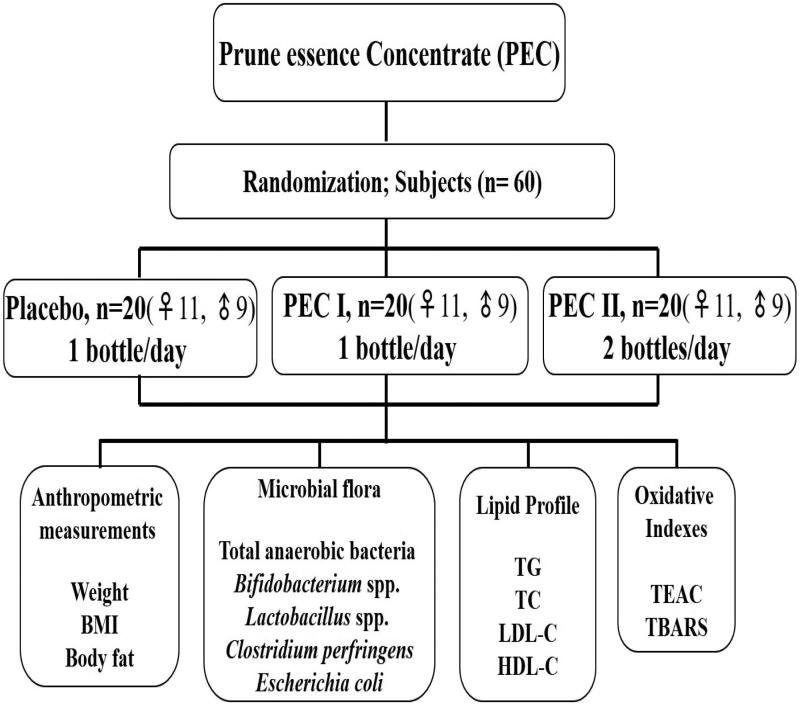
The schematic representation of the present study.

The effect of PEC on anthropometric parameters in healthy mild hypercholesterolemic subjects is summarized in Table 1. No substantial alterations were observed in any of the levels of anthropometric parameters, such as body weight, BMI and body fat in test (PEC I and II) or placebo-ingested subjects. However, a slight decrease in BMI and body weight was noted in both test groups in the follow-up period, but not significant. The schematic representation of the present study is portrayed in Figure 1.

**Table 1.** The effect of PEC on anthropometric parameters in healthy mild hypercholesterolemic subjects.

Weeks	Groups	Weight (kg)	BMI (kg/m^2^)	Body fat (%)
Initial	Placebo	64.29 ± 13.21^a^	23.59 ± 4.04^a^	25.99 ± 5.80^a^
	PEC I	59.88 ± 11.03^a^	21.57 ± 2.45^a^	24.25 ± 4.14^a^
	PEC II	63.50 ± 12.60^a^	22.82 ± 2.98^a^	25.99 ± 4.64^a^
4th Week	Placebo	63.99 ± 13.66^a^	23.48 ± 4.24^a^	25.16 ± 4.00^a^
	PEC I	59.65 ± 10.75^a^	21.49 ± 2.33^a^	24.39 ± 4.85^a^
	PEC II	63.60 ± 12.70^a^	23.02 ± 2.97^a^	25.67 ± 4.66^a^
Follow-up (6th week)	Placebo	63.89 ± 11.34^a^	23.62 ± 4.08^a^	25.56 ± 5.31^a^
	PEC I	60.01 ± 11.86^a^	21.25 ± 2.48^a^	24.17 ± 4.22^a^
	PEC II	63.31 ± 12.50^a^	22.90 ± 2.68^a^	25.93 ± 4.73^a^

Values are expressed as means ± SD. Data within the same group bearing different superscripts letters were significantly different (*p* < 0.05). BMI: Body mass index.

Table 2 exemplifies the effect of PEC on the intestinal microflora in healthy mild hypercholesterolemic subjects. Four weeks of treatment with PEC (I and II) exhibited a selective escalation in the intestinal beneficial bacterial population, especially *Bifidobacterium*, *Lactobacillus* spp. and total anaerobic bacterial count on equivalence with the initial value. In comparison with placebo group the count of *Bifidobacterium*, *Lactobacillus* spp. and the total anaerobic bacterial count was substantially elevated at the end of the intervention (4th week). Meanwhile, the harmful bacteria like *C. perfringens* and *E. coli* were greatly restrained upon treatment with PEC. However, during the follow-up (6th week) *C. perfringens* and *E. coli* were slightly increased, whereas *Bifidobacterium*, *Lactobacillus* spp. and the total anaerobic bacterial count was markedly reduced due to stoppage of PEC treatment.

**Table 2.** The effect of PEC on the intestinal microflora count in healthy mild hypercholesterolemic subjects.

Weeks	Groups	*E. coli* (CFU/g)	*C. perfringens* (CFU/g)	*Lactobacillus* spp (CFU/g)	*Bifidobacterium* spp (CFU/g)	TAB (CFU/g)
Initial	Placebo	7.79 ± 0.43^a^	8.03 ± 0.31^a^	8.24 ± 0.93^a^	7.99 ± 0.66^a^	9.60 ± 0.32^a^
	PEC I	7.99 ± 0.28^a^	8.03 ± 0.43^a^	8.09 ± 0.51^c^	7.06 ± 0.53^c^	9.36 ± 0.30^b^
	PEC II	7.67 ± 0.20^a^	8.02 ± 0.44^a^	8.09 ± 0.69^c^	7.52 ± 0.64^c^	9.25 ± 0.70^b^
2nd Week	Placebo	7.77 ± 0.48^a^	8.03 ± 0.30^a^	8.35 ± 0.52^a^	8.02 ± 0.72^a^	9.66 ± 0.41^a^
	PEC I	7.75 ± 0.30^a^	7.79 ± 0.42^b^	8.24 ± 1.39^b^	7.94 ± 0.59^b^	9.65 ± 0.29^a,b^
	PEC II	7.28 ± 0.25^b,^*	7.67 ± 0.42^b,^*	8.93 ± 0.66^b,^*	8.61 ± 0.61^a,^*	9.75 ± 0.93^a,b^
4th Week	Placebo	7.80 ± 0.43^a^	8.02 ± 0.31^a^	8.25 ± 0.97^a^	8.07 ± 0.86^a^	9.64 ± 0.42^a^
	PEC I	7.49 ± 0.27^b,^*	7.55 ± 0.41^c,^*	8.66 ± 0.86^a,^*	8.37 ± 0.65^a^	9.95 ± 0.34^a^
	PEC II	6.95 ± 0.30^c,^**	7.35 ± 0.38^b,^*	9.41 ± 0.48^a,^**	8.98 ± 0.53^a,^*	10.21 ± 0.43^a,^*
Follow-up (6th week)	Placebo	7.77 ± 0.43^a^	8.21 ± 0.29^a^	8.46 ± 0.56^a^	8.00 ± 0.87^a^	9.68 ± 0.50^a^
	PEC I	7.93 ± 0.24^a^	8.17 ± 0.46^a^	8.02 ± 0.42^c^	7.37 ± 0.43^b,c^	9.30 ± 0.32^b^
	PEC II	7.54 ± 0.23^a^	8.16 ± 0.38^a^	8.39 ± 0.41^b^	7.95 ± 0.50^c^	9.39 ± 0.19^b^

Values are expressed as means ± SD. Data within the same group bearing different superscripts letters were significantly different (*p* < 0.05).

* *p <* 0.05,

** *p <* 0.01 (Placebo vs PEC I and II in 2nd and 4th week). TAB: Total anaerobic bacteria.

Table 3 represents the effect of PEC on lipid profile in healthy mild hypercholesterolemic subjects. PEC (I and II) ingested subjects showed a notable decrease (*p* < 0.05) in the levels of TC, LDL-c with a minor improvement in the levels of HDL-c as compared with baseline (0 week). After 4 weeks of intervention, the levels of both TC and LDL-c in PEC I and II were concomitantly decreased in comparison with the placebo group. Nevertheless, during the follow-up period, the levels of TC and LDL-c were mildly elevated (Table 2). In the case of TG in any of the group did not infer any changes.

**Table 3.** The effect of PEC on the plasma lipid profile in healthy mild hypercholesterolemic subjects.

Weeks	Groups	TG (mg/dL)	TC (mg/dL)	LDL (mg/dL)	HDL (mg/dL)
Initial	Placebo	82.60 ± 16.31^a^	179.10 ± 27.43^a^	99.30 ± 20.20^a^	58.95 ± 8.06^a^
	PEC I	78.20 ± 13.67^a^	173.45 ± 24.83^a^	98.80 ± 26.10^a^	63.70 ± 8.66^a^
	PEC II	85.45 ± 23.93^a^	179.40 ± 31.47^a^	104.10 ± 30.36^a^	58.80 ± 9.97^a^
4th Week	Placebo	86.80 ± 18.40^a^	174.70 ± 22.59^a^	98.46 ± 18.36^a^	57.30 ± 7.86^a^
	PEC I	79.60 ± 15.12^a^	163.20 ± 28.24^b,^*	92.20 ± 25.67^b^*	65.65 ± 8.88^a^
	PEC II	88.21 ± 21.34^a^	166.85 ± 28.45^b,^**	98.34 ± 27.50^b,^*	59.70 ± 10.67^a^
Follow-up (6th week)	Placebo	85.01 ± 21.04^a^	176.23 ± 24.45^a^	99.23 ± 19.41^a^	57.05 ± 9.05^a^
	PEC I	78.45 ± 14.94^a^	169.29 ± 29.29^a^	96.45 ± 22.56^a^	64.70 ± 6.01^a^
	PEC II	87.35 ± 20.25^a^	171.67 ± 30.63^a^	103.73 ± 21.82^a^	60.10 ± 9.66^a^

Values are expressed as means ± SD. Data within the same group bearing different superscripts letters were significantly different (*p* < 0.05).

* *p <* 0.05,

** *p <* 0.01 (Placebo vs PEC I and II in 4th week).

The efficacy of PEC on the oxidative indexes in healthy mild hypercholesterolemic subjects is summarized in Table 4. In equivalence with placebo, PEC-consumed subjects showed a considerable augmentation in the levels of TEAC with a substantial alleviation (*p* < 0.05) in TBARS levels. Four weeks of treatment with PEC I and II showed considerable changes in the concentration of TBARS and TEAC compared with the baseline. There were no substantial changes observed in the placebo group for both TEAC and TBARS. Overall, both the PEC groups showed notable changes on equivalence with placebo. However, PEC II showed much better results than PEC I, but no significant difference was noted among those groups.

**Table 4.** The effect of FPE on the oxidative indexes in healthy mild hypercholesterolemic subjects.

Weeks	Groups	TEAC (mM)	TBARS (μM)
Initial	Placebo	0.41 ± 0.02^a^	0.82 ± 0.14^a^
	PEC I	0.40 ± 0.05^a^	0.82 ± 0.21^a^
	PEC II	0.40 ± 0.07^a^	0.81 ± 0.18^a^
4th Week	Placebo	0.41 ± 0.06^a^	0.83 ± 0.11^a^
	PEC I	0.43 ± 0.05^a^	0.80 ± 0.13^b^
	PEC II	0.43 ± 0.06^a^	0.69 ± 0.07^c,^*
Follow-up (6th week)	Placebo	0.42 ± 0.03^a^	0.81 ± 0.09^a^
	PEC I	0.41 ± 0.06^a^	0.81 ± 0.08^a^
	PEC II	0.42 ± 0.05^a^	0.74 ± 0.11^b^

Values are expressed as means ± SD. Data within the same group bearing different superscripts letters were significantly different (*p* < 0.05).

* *p <* 0.05 (Placebo vs PEC I and II in 4th week).

## Discussion

Several pre-clinical and clinical studies suggested that consumption of dried plum (prunes) extracts or concentrate suppressed the cholesterol level (hypocholesterolemic property) as well as lowered the LDL-oxidation, and thus attenuated the incidence of CVDs (Tinker et al. 1991; Stacewicz-Sapuntzakis et al. 2001; Stacewicz-Sapuntzakis 2013). Nevertheless, the exact association is yet to prove and also the clinical trial results are inconclusive. Hence, the present study was framed to assess whether consumption of PEC might alter the intestinal microflora and contributed to health-promoting activities like hypocholesterolemic and antioxidant activities. The HPLC fingerprinting data revealed the presence of various phenolic acids like chlorogenic acid, neochlorogenic acid and cryptochlorogenic acid (data not shown) and those beneficial effects of PEC might be due to those phenolic acids. Similarly, Putnam et al. (2007) demonstrated that chlorogenic and neochlorogenic acids are the crucial polyphenols which contribute to several biological functions in prune extract.

The anthropometric analysis was evaluated to confirm physiological changes related to body composition, after the administration of PEC. Intake of PEC I and II did not show any notable changes in the values of body weight, BMI and body fat. During the follow-up period (6th week), a minor decrement was observed in the levels of BMI and body weight, but the changes were not significant. The outcome of anthropometric analysis clearly demonstrated that ingestion of PEC for four weeks did not alter the body morphology or composition, and it was safe to consume prune concentrate without any adverse events. Likewise, the placebo group also did not showcase any changes in any of the anthropometric measurements.

The intestinal/gut microflora is a complex community of microorganisms (probiotics) such as bacteria, viruses, and protozoan, which reside in gastric tracts and feeds on undigested carbohydrates (prebiotics). The intestinal bacterial community is discriminated into beneficial and harmful bacteria (Fujimura et al. 2010). *Bifidobacterium* spp. and *Lactobacillus* spp. genera are classified as beneficial bacteria, since they render various health-promoting function by the production of short-chain fatty acids (SCFA) and vitamins, helping digestion and absorption of nutrients, immune-stimulant, inhibiting the growth of harmful bacteria or pathogens, lowering cholesterol and ammonia levels. Whereas, *Clostridium perfringes* spp. and *E. coli* are considered as harmful bacteria, which favour deleterious effects to human (Vendrame et al. 2011).

PEC (I and II)-consumed subjects showed a remarkable improvement in the population of beneficial bacteria’s community, especially *Bifidobacterium*, *Lactobacillus* spp., and total anaerobic bacterial count on comparison with the baseline. It might be due to the presence of both soluble (pectin) and insoluble dietary fibers in PEC, along with sugar alcohols such as sorbitol and xylitol as well as oligosaccharide (inulin, xylo and fructo-oligosaccharide). These compounds were reported to act as prebiotics and thus promoting the growth of beneficial bacteria in colon through suppressing the intestinal pH, elevated production of SCFA, also stimulated gastric emptying (giving bulkiness to feces thus decreased bowel transit time), as well as decrease the intestinal cholesterol absorption (Fujimura et al. 2010; Alonso & Guarner 2013; Stacewicz-Sapuntzakis 2013). While, the harmful bacteria like *C. perfringens* and *E. coli* were markedly abolished upon supplementation with PEC, owing to the counteracting property of the beneficial bacterial community to balance the intestinal microbiota. Furthermore, chlorogenic acid present in prunes may be metabolized by the intestinal microbiota to produce caffeic acid, which favors the proliferation of *Bifidobacterium* spp. and subsequently, *Lactobacillus* spp as well (Parkar et al. 2013). Noratto et al. (2014) observed that intake of plum juice significantly improvement in the content of beneficial bacteria such as *Lactobacillus* and *Bifidobacterium* spp. in obese Zucker rats. Nevertheless, during the follow-up (6th week) *C. perfringens* and *E. coli* were slightly increased, whereas *Bifidobacterium*, *Lactobacillus* spp., and the total anaerobic bacterial count was markedly reduced due to stoppage of PEC consumption. In addition, PEC intake also exponentially elevated the faecal weight and thus lowered the intestinal bowel transit time owing to increased insoluble fibres (gives bulkiness to faeces). Therefore, prune is highly prescribed for constipation subjects, since it acts as a laxative agent (data are given as Supplementary file).

Hypercholesterolemia (elevated serum total cholesterol) is believed as one of the major risk factors for CVDs (Lu et al. 2015). Several experimental studies had hinted that lowering of lipid levels probably reduced the prevalence of CVDs. Therefore, to evaluate the hypocholesterolemic efficacy of PEC, various lipid parameters like TG, TC, LDL-c, HDL-c were quantified. Intake of PEC (I and II) remarkably lowered the levels of TC, LDL-c with a mild increase in the levels of HDL-c as compared with baseline (0 week). However, on the 4th week of intervention, PEC (I and II) group presented lesser levels of both TC and LDL-c in comparison with the placebo group. Stacewicz-Sapuntzakis et al. (2001, 2013) also highlighted that pectin (fruit-soluble fiber) present in the prune could lower the cholesterol level through enhancing bile acid excretion, and thus deviate the excessive cholesterol levels to bile acid synthesis. In addition, pectin may also increase the viscosity of mucus in the gastrointestinal (GI) tract and thereby decrease the cholesterol absorption (Sriamornsak 2003). Moreover, phytosterols of prunes are shown to exert hypocholesterolemic activity, by upregulating the mRNA expression of LDL-receptor (Igwe & Charlton 2016). Nevertheless, during the follow-up period, the levels of TC and LDL-c were moderately elevated. However, the levels of TG in any of the group did not show any significant changes. Our results were in concordance with the results of Howarth et al. (2010), who demonstrated that consumption of dried plum, did not alter the TG levels.

Ample amount of studies indicated that excessive free radicals are generated during hypercholesterolemic condition and results in oxidative stress (imbalance in free radicals and antioxidants) and subsequently results in CVDs (Chiu et al. 2015; Lu et al. 2015). Therefore, during the present study various oxidative indexes were evaluated to check the antioxidant activity of PEC. PEC ingested subjects displayed a concomitant reduction in the levels of TEAC with a substantial decline in the levels of TBARS as with the placebo group. Consumption of dried plum snack (prune) for two weeks in healthy women showed a pronounced elevation in the levels of serum TEAC activity (Kaper et al. 2010). As pointed out earlier, prunes contain a high amount of chlorogenic, cryptochlorogenic and neochlorogenic acids, which probably bestowed to increased antioxidant capacity and thus lower the lipid peroxidation products such as TBARS (De Gonzalez et al. 2008). In addition, prune extracts are proven to suppress the LDL-oxidation process, thereby decrease the risk of CVD, owing to its antioxidant activity (Gallaher & Gallaher 2009). However, PEC II showed much better results than PEC I, but no significant difference was noted among those groups.

Lever et al. (2014) in his review also demonstrated that intake of prune might favour the intestinal microflora count and hence exhibit several health promoting factors such as antioxidant, anti-inflammatory, anti-diabetic, as well as a hypocholesterolemic agents. The results of our studies also proved that PEC could effectively attenuate the levels of TEAC and beneficial bacteria such as *B.* spp *and L.* spp. count, with decreased TC and LDL-c, as well as harmful bacteria species, when compared to the placebo group. Therefore, we suggest that PEC could improve the intestinal microflora and thus favours the hypocholesterolemic and antioxidant properties. Limitations of the current study were not to evaluate the mechanism behind the improvement of beneficial bacteria, as well as the lack of flow cytometric analysis, and genomic changes of all those bacteria would hamper our results for not elaborating the exact interaction with PEC.

## Conclusions

The current finding suggests that consumption of both PEC I and II for four weeks considerably abolished TC, LDL-c, TBARS and harmful bacteria like *E. coli and C. perfringens* as well as substantially attenuated the levels of TEAC and beneficial bacteria such as *Bifidobacterium* spp and *Lactobacillus* spp. count. Although both PEC showed good results but still PEC II showed slightly better hypocholesterolemic and antioxidant activity than PEC I. Therefore, we concluded that PEC intake could positively alter the human intestinal flora and thereby enhance various physiological functions and favour various health benefits. In future, the active components of PEC can be isolated and tested for its hypocholesterolemic property.

## Supplementary Material

Wang_Chin_et_al_supplemental_content.zip

## References

[CIT0001] Alonso VR, Guarner F. 2013 Linking the gut microbiota to human health. Br J Nutr. 109:S21–SS6. 23360877 10.1017/S0007114512005235

[CIT0002] Beerens H. 1991 Detection of bifidobacteria by using propionic acid as a selective agent. Appl Environ Microbiol. 57:2418 1768114 10.1128/aem.57.8.2418-2419.1991 PMC183588

[CIT0003] Chiu HF, Shen YC, Huang TY, Venkatakrishnan K, Wang CK. 2015 Cardioprotective eefficacy of red wine extract of onion in healthy hypercholesterolemic subjects. Phytother Res. 30:380–385. 26631904 10.1002/ptr.5537

[CIT0004] De Gonzalez MN, Hafley BS, Boleman RM, Miller RK, Rhee KS, Keeton JT. 2008 Antioxidant properties of plum concentrates and powder in precooked roast beef to reduce lipid oxidation. Meat Sci. 80:997–1004. 22063828 10.1016/j.meatsci.2008.04.014

[CIT0005] Draper H, Hadley M. 1990 Malondialdehyde determination as index of lipid peroxidation. Meth Enzymol. 186:421–431. 2233309 10.1016/0076-6879(90)86135-i

[CIT0006] El-Gawad IAA, El-Sayed E, Hafez S, El-Zeini H, Saleh F. 2005 The hypocholesterolaemic effect of milk yoghurt and soy-yoghurt containing bifidobacteria in rats fed on a cholesterol-enriched diet. Int Dairy J. 15:37–44.

[CIT0007] Fujimura KE, Slusher NA, Cabana MD, Lynch SV. 2010 Role of the gut microbiota in defining human health. Expert Rev Anti Infect Ther. 8:435–454. 20377338 10.1586/eri.10.14 PMC2881665

[CIT0008] Gallaher CM, Gallaher DD. 2009 Dried plums (prunes) reduce atherosclerosis lesion area in apolipoprotein E-deficient mice. Br J Nutr. 101:233–239. 18761779 10.1017/S0007114508995684

[CIT0009] Harmon SM, Kautter DA, Peeler JT. 1971 Comparison of media for the enumeration of *Clostridium perfringens*. Appl Microbiol. 21:922–927. 4324885 10.1128/am.21.5.922-927.1971 PMC377309

[CIT0010] Howarth L, Petrisko Y, Furchner-Evanson A, Nemoseck T, Kern M. 2010 Snack selection influences nutrient intake, triglycerides, and bowel habits of adult women: a pilot study. J Am Diet Assoc. 110:1322–21327. 20800123 10.1016/j.jada.2010.06.002

[CIT0011] Igwe EO, Charlton KE. 2016 A systematic review on the health effects of plums (*Prunus domestica* and *Prunus salicina*). Phytother Res. 30:701–731. 26992121 10.1002/ptr.5581

[CIT0012] Kaper S, Howarth LS, Petrisko Y, Furchner-Evanson A, Nemoseck T, Hong MY, Kern M. 2010 Dried plums consumed twice daily increase antioxidant capacity after two weeks in adult women. FASEB J. 24:564–565.

[CIT0013] Lever E, Cole J, Scott SM, Emery PW, Whelan K. 2014 Systematic review: the effect of prunes on gastrointestinal function. Aliment Pharmacol Ther. 40:750–758. 25109788 10.1111/apt.12913

[CIT0014] Lu TM, Chiu HF, Shen YC, Chung CC, Venkatakrishnan K, Wang CK. 2015 Hypocholesterolemic efficacy of quercetin rich onion juice in healthy mild hypercholesterolemic adults: a pilot study. Plant Foods Hum Nutr. 70:395–400. 26385226 10.1007/s11130-015-0507-4

[CIT0015] Manafi M, Kneifel W. 1989 A combined chromogenic-fluorogenic medium for the simultaneous detection of *coliform groups* and *E. coli* in water. Int J Hyg Environ Med. 189:225–234. 2697207

[CIT0016] Miller NJ, Rice-Evans C, Davies MJ, Gopinathan V, Milner A. 1993 A novel method for measuring antioxidant capacity and its application to monitoring the antioxidant status in premature neonates. Clin Sci. 84:407–412. 8482045 10.1042/cs0840407

[CIT0017] Noratto GD, Garcia-Mazcorro JF, Markel M, Martino HS, Minamoto Y, Steiner JM, Byrne D, Suchodolski JS, Mertens-Talcott SU. 2014 Carbohydrate-free peach (*Prunus persica*) and plum (*Prunus domestica*) juice affects fecal microbial ecology in an obese animal model. PLoS One. 9:e101723. 25007331 10.1371/journal.pone.0101723 PMC4090149

[CIT0018] Oyebode O, Gordon-Dseagu V, Walker A, Mindell JS. 2014 Fruit and vegetable consumption and all-cause, cancer and CVD mortality: analysis of Health Survey for England data. J Epidemiol Community Health. 68:856–862. 24687909 10.1136/jech-2013-203500 PMC4145465

[CIT0019] Parkar SG, Trower TM, Stevenson DE. 2013 Fecal microbial metabolism of polyphenols and its effects on human gut microbiota. Anaerobe. 23:12–19. 23916722 10.1016/j.anaerobe.2013.07.009

[CIT0020] Putnam SE, Scutt AM, Bicknell K, Priestley CM, Williamson EM. 2007 Natural products as alternative treatments for metabolic bone disorders and for maintenance of bone health. Phytother Res. 21:99–112. 17106868 10.1002/ptr.2030

[CIT0021] Qiao Y, Sun J, Xia S, Tang X, Shi Y, Le G. 2014 Effects of resveratrol on gut microbiota and fat storage in a mouse model with high-fat-induced obesity. Food Funct. 5:1241–1249. 24722352 10.1039/c3fo60630a

[CIT0022] Rogosa M, Mitchell JA, Wiseman RF. 1951 A selective medium for the isolation and enumeration of oral and fecal lactobacilli. J Bacteriol. 62:132 14861168 10.1128/jb.62.1.132-133.1951 PMC386093

[CIT0023] Scheid MMA, Moreno YMF, Junior MRM, Pastore GM. 2013 Effect of prebiotics on the health of the elderly. Food Res Int. 53:426–432.

[CIT0024] Slavin JL, Lloyd B. 2012 Health benefits of fruits and vegetables. Adv Nutr. 3:506–516. 22797986 10.3945/an.112.002154 PMC3649719

[CIT0025] Sriamornsak P. 2003 Chemistry of pectin and its pharmaceutical uses: a review. Silpakorn Univ Int J. 3:206–228.

[CIT0026] Stacewicz-Sapuntzakis M, Bowen PE, Hussain EA, Damayanti-Wood BI, Farnsworth NR. 2001 Chemical composition and potential health effects of prunes: a functional food? Crit Rev Food Sci Nutr. 41:251–286. 11401245 10.1080/20014091091814

[CIT0027] Stacewicz-Sapuntzakis M. 2013 Dried plums and their products: composition and health effects-an updated review. Crit Rev Food Sci Nutr. 53:1277–1302. 24090144 10.1080/10408398.2011.563880

[CIT0028] Teo K, Lear S, Islam S, Mony P, Dehghan M, Li W, Rosengren A, Lopez-Jaramillo P, Diaz R, Oliveira G, et al 2013 Prevalence of a healthy lifestyle among individuals with cardiovascular disease in high-, middle-and low-income countries: the Prospective Urban Rural Epidemiology (PURE) study. JAMA. 309:1613–1621. 23592106 10.1001/jama.2013.3519

[CIT0029] Tinker LF, Schneeman BO, Davis PA, Gallaher DD, Waggoner CR. 1991 Consumption of prunes as a source of dietary fiber in men with mild hypercholesterolemia. Am J Clin Nutr. 53:1259–1265. 1850578 10.1093/ajcn/53.5.1259

[CIT0030] Vendrame S, Guglielmetti S, Riso P, Arioli S, Klimis-Zacas D, Porrini M. 2011 Six-week consumption of a wild blueberry powder drink increases bifidobacteria in the human gut. J Agric Food Chem. 59:12815–12820. 22060186 10.1021/jf2028686

