# Supplementary material for: Regulatory/modulatory effect of prune essence concentrate on intestinal function and blood lipids
Source: Pharm Biol. 2017 Feb 5;55(1):974–9. doi: 10.1080/13880209.2017.1285323 (PMC6130511; doi:10.1080/13880209.2017.1285323)
Supplement: Wang_Chin_et_al_supplemental_content.zip [file IPHB_A_1285323_SM2017.zip › Wang Chin et al supplemental content.pdf]

Supplementary Data

The weight of feces (gram) of subjects

Weeks	Placebo	PEC I	PEC II
**Initial**	115.49±39.62[a]	111.08±52.99[a]	101.84±37.93[a]
**2nd week**	117.20±38.96[a]	171.93±76.31[b]	167.00±45.79[b]
**4th week**	117.68±61.56[a]	173.99±92.77[b**]	220.83±76.17[c**]
**6th week**	116.60±45.23[a]	130.81±57.29[ab]	138.47±53.72[ab]

Data are expressed as mean ± SD. Data within the same column bearing different superscript letters were significantly different (P< 0.05). * *P* <0.05, ** *P* <0.01 (Placebo vs PEC I and II in 4th week).

The time (min) for passing through intestine/ Bowel transit time

Weeks	Placebo	PEC I	PEC II
**Initial**	117.80±48.83[a]	118.15±29.06[a]	118.25±27.98[a]
**4th week**	115.70±51.72[a]	91.80±26.26[b]	81.35±19.78[b]
**6th week**	116.45±24.67[a]	98.56±15.35[b]	87.13±14.45[b]

Data are expressed as mean ± SD. Data within the same column bearing different superscript letters were significantly different (P< 0.05). * *P* <0.05, ** *P* <0.01 (Placebo vs PEC I and II in 4th week).

For Peer Review Only
